# Alcohol Use among Older Adults: SABE Cohort Study, São Paulo, Brazil

**DOI:** 10.1371/journal.pone.0085548

**Published:** 2014-01-08

**Authors:** Gabriela Arantes Wagner, Maria Lucia Lebrão, Yeda Aparecida de Oliveira Duarte, Dirce Maria Trevisan Zanetta

**Affiliations:** 1 Department of Epidemiology, School of Public Health, University of São Paulo, São Paulo Brazil; 2 Medical-Surgical Nursing Department, School of Nursing, University of São Paulo, São Paulo, Brazil; Universidade Federal do Acre (Federal University of Acre), Brazil

## Abstract

In recent years, Brazil has demonstrated a new demographic pattern characterized by a reduction in both birth and mortality rates and a significant increase in the number of older adults. The purpose of the present study was to describe the frequency of alcohol intake in a representative sample community of older adults in the city of São Paulo, Brazil, followed over a six-year period. A prospective *Saúde, Bem-Estar e Envelhecimento* (SABE [Health, Wellbeing and Aging]) cohort study conducted in 2000 and 2006 in City of São Paulo, Brazil. 2,143 individuals aged 60 years or older selected through multi-stage sampling in the year 2000 (41.4% male and 58.6% women) and 1,115 individuals belonging to the follow-up cohort evaluated in 2006. The frequency of alcohol intake in the previous three months was obtained through self-reports of interviewees. The results demonstrate that in 2000, alcohol consumption was less than one day a week among 79.7% of the sample, one to three days a week among 13.0% and four or more days a week among 7.3%. In agreement with findings on other populations, consumption four or more days a week was more frequent among the male gender as well as those with greater schooling and income and good self-rated health (p<0.05). The longitudinal analysis demonstrated an increase in the frequency of alcohol consumption one to three times a week among the individuals in the 2006 follow-up study. In the present population-based sample, alcohol intake was low and the frequency of moderate alcohol consumption increased over the years. The present study can assist understanding the changes in alcohol intake among older adults throughout time and the ageing process.

## Introduction

In recent years, Brazil has demonstrated a new demographic pattern characterized by a reduction in both birth and mortality rates, with profound transformations in the composition of the age structure of the population and a significant increase in the number of older adults. Health problems associated with the use of alcohol and other drugs among older adults are generally related to physiological changes stemming from the ageing process, such as increased sensitivity to such substances, an increase in the number of comorbidities and the concomitant use of various medications. On the other hand, the moderate use of alcohol among older adults may be related to a reduction in the mortality rate due to heart disease and related outcomes [1]–[13].

While studies report a tendency toward a reduction in alcohol use with the advance in age, this use may be underestimated, as alcohol-related problems among older adults may be clinically silent or atypical. Moreover, drinking may be considered a shameful behavior by some individuals, which could lead to information bias regarding alcohol intake in epidemiological surveys [14], [15].

Both cross-sectional and longitudinal studies on alcohol intake among older adults have been carried out in different countries to characterize and understand possible changes in patterns of use and associations with demographic, health and behavioral factors. The understanding of these factors is important to the development of adequate public policies [3].

Little is known regarding the frequency of alcohol consumption among the elderly in Brazil, or how these individuals change their alcohol intake over time [16], [17]. Thus, the aims of the present study were to 1) describe the frequency of alcohol intake in the previous three months in a representative sample of older adults in the city of São Paulo in the year 2000, 2) determine associations with socio-demographic and health-related factors, and 3) analyze possible changes in the frequency of alcohol intake after a six-year follow-up period in comparison to baseline and the associations of baseline characteristics with the increase of alcohol consumption among the elderly.

## Methods

### Ethical Considerations

This study received approval from the Human Research Ethics Committee of the School of Public Health, University of São Paulo, Brazil (CONEP 315/99 and 83/06). Participation was voluntary and a signed statement of informed consent was obtained from all participants [18].

### Subjects

**Figure 1:**
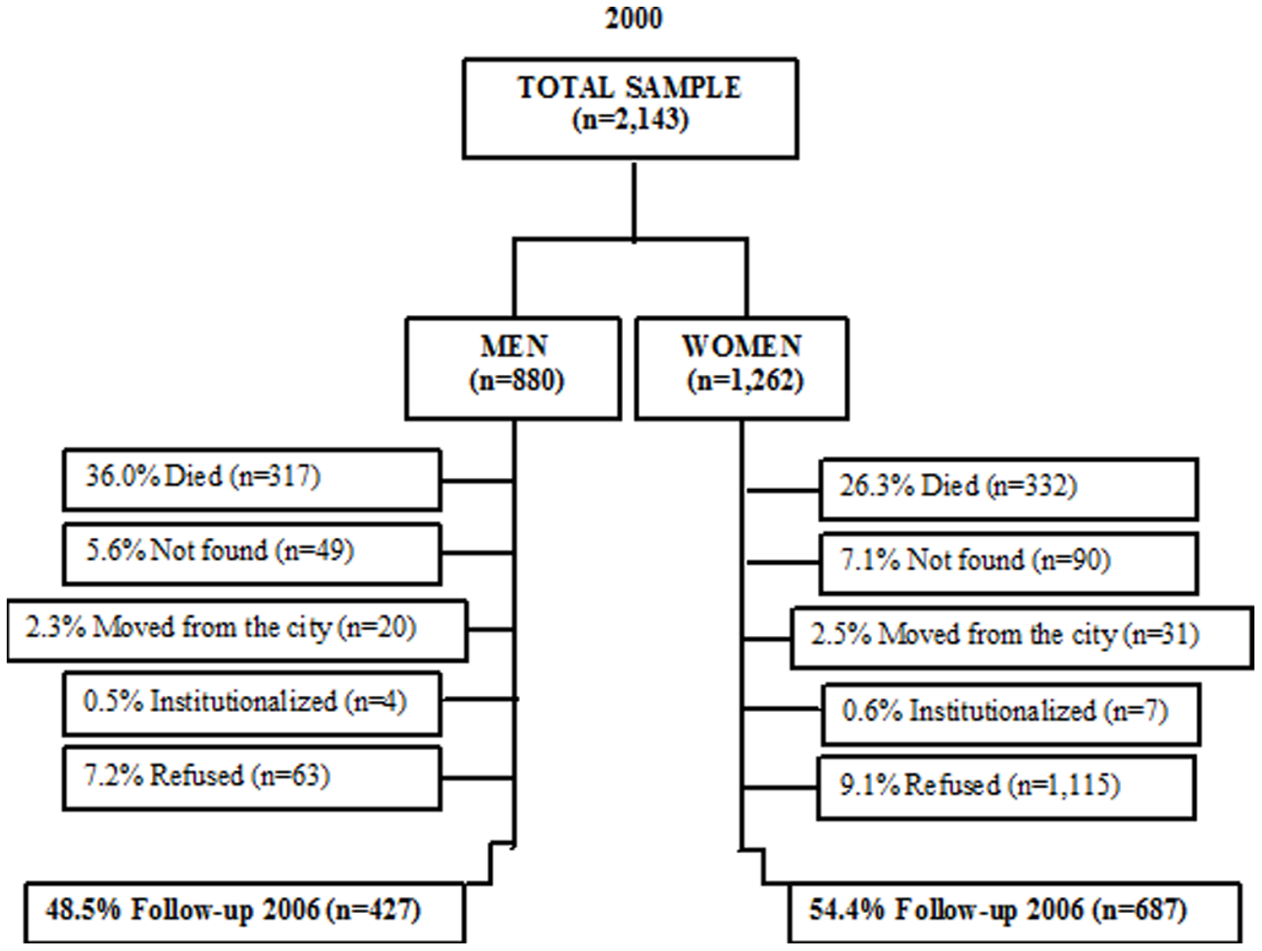
Algorithm: Study Design (2000–2006).

A prospective longitudinal study was carried out with data from the *Saúde, Bem-Estar e Envelhecimento* (SABE [Health, Wellbeing and Aging]) cohort study. The first wave of the study was conducted in the city of São Paulo from 2000 to 2001 and involved 2,143 community-dwelling individuals aged 60 years and older selected through multiple-stage sampling, as described elsewhere [18]. In 2006, a second wave was conducted. During this follow-up study, 1,115 of the elderly individuals who had participated in the baseline study were located and agreed to undergo a new set of interviews involving the same procedures. Among those lost to follow up, 649 had died, 139 were not found, 51 had moved from the city, 11 were institutionalized and 178 refused to participate in the continuation of the study (Figure 1). Data were collected by an interviewer-administered questionnaire at the participants’ homes and included information on socio-demographic factors, general health and living conditions. The results of the cognitive status were used as a filter for evaluating autonomy of the elderly; with the purpose of minimize recall and other bias. For assessment of cognitive function, Mini Mental State Examination (MMSE) was validated for the SABE Study, due to the low level of schooling of the South American elderly population. This measure has thirteen items that are less dependent upon schooling and the cut-off point is a score of 12 or less. Elderly with score below 12 were assisted by a proxy respondent [19], [20].

### Variables

The frequency of alcohol intake was evaluated based on consumption in the three months prior to the interview in both 2000 and 2006, as determined by self-reports of the individuals in response to the following question: *“In the last three months, how many days a weeks, on average, have you had alcoholic beverages (for instance, beer, wine, liquor or other drinking containing alcohol)?”* For statistical purposes, intake was classified in three frequency categories: low intake (< one day a week); moderate intake (one to three days a week) and high intake (four or more days a week).

The following variables collected at baseline were also analyzed: gender (male/female); age in years (60 to 64, 65 to 74 and 75 or older), schooling in completed years of study (<1 to 3 years, 4 to 7 years and 8 or more years), income categorized by quartiles; marital status (married/stable union, widowed or single/divorced/separated); ethnic background (Caucasian, African descent/mulatto or other); self-rated health (good/poor to fair); number of chronic diseases (none, 1 or ≥2); history of falls in previous 12 months (yes/no); and smoking habit (non-smoker, ex-smoker or current smoker).

### Statistical Analysis

Descriptive analysis was carried out for the socio-demographic characteristics of the sample and frequency of alcohol intake in the year 2000 (baseline). Comparisons were made using Pearson’s chi-square (χ^2^) test with the Rao-Scott correction [21]. The analysis incorporated weights to correct for the different selection probabilities of the participants and the results are expressed as weighted values. All p-values<0.050 were considered statistically significant. The Survey package of the R 2.13.1 program was used [22], which offers procedures for the analysis of complex sample inquiries and allows the incorporation of the different weights of the observations that influence the parameter estimates of the total population and the effect of sampling on the variance estimates. Polynomial regression was used for the analysis of factors associated with alcohol intake, with alcohol use in the previous three months as the dependent variable. The reference category for this analysis was low intake, contrasting with moderate and high intake. Baseline variables with a p-value<0.200 in the univariate analysis were selected for multiple polynomial model, for which the forward stepwise method was used. In the model, the magnitude of the associations was estimated using odds ratios and respective 95% confidence intervals (95% CI). Variables non-significant were excluded, if there was no modification greater than 10% in other parameters estimated.

After the six-year follow-up period, changes in alcohol intake in comparison to the baseline evaluation were evaluated by estimating the frequency of individuals who referred same intake as in baseline, categorized as individuals with stable low intake (less than once a week) or stable drinkers (once a week or more), those who referred increased or decreased the alcohol intake. Polynomial regression was used for the analysis of factors of baseline characteristics associated with increase or sustained alcohol intake in the follow-up. The reference category for this analysis was stable low intake. Associations with a p-value<0.200 in the univariate analysis contrasting stable low intake, increased intake and stable drinkers, were incorporated into the multiple polynomial model by forward procedure. Falls in the last 12 months was significant in the univariate analysis but was not included in the modelling, as we considered this variable to be consequence and not possible risk factor for alcohol intake. The magnitude of the associations was estimated using odds ratios and respective 95% confidence intervals. Variables non-significant were excluded, if there was no modification greater than 10% in other estimated parameters.

To evaluate the cohort effect of a birth date difference of six years, the frequencies of alcohol use and 95% confidence intervals were estimated by age group of the individuals evaluated in 2006 and compared with the findings in the same age groups in 2000.

## Results

A greater proportion of the sample was female (58.6%). Regarding alcohol intake in the previous three months, 79.7% (95% CI = 77.0%–82.1%) of the individuals drank less than once a week, 13.0% (95% CI = 11.0%-to 15.3%) drank one to three times a week and 7.3% (95% CI = 6.2%–8.7%) drank four or more times a week. Table 1 display the characteristics of the older adults interviewed in 2000 for both genders. Compared with women, men had greater income, more were married, and had higher schooling. Most of the men were ex-smokers or current smokers, and most of the women were never smokers. The prevalence of multimorbidity (more than 2 chronic diseases) was higher among women. A greater percentage of both genders drank<1 day a week.

**Table 1:** Weighted distribution of alcohol intake in previous three months and baseline characteristics by gender in adults aged 60 and older: 2000 *Saúde, Bem-Estar e Envelhecimento* (SABE [Health, Wellbeing and Aging]) Survey.

		MEN			WOMEN		
BASELINECHARACTERISTICS		Weighted%#	Weightedn&	Unweightedn$	Weighted%#	Weightedn&	Unweightedn$
Age	60–64	34.3	118,694	171	30.9	151,576	256
	65–74	46.8	161,840	274	44.8	219,386	449
	≥75	18.9	65,151	435	24.2	118,297	557
Income	1st quartile	24.6	77,719	257	56.6	208,269	592
	2nd quartile	14.5	45,878	129	12.8	47,189	129
	3rd quartile	32.5	102,535	242	16.0	58,970	153
	4th quartile	28.4	89,725	195	14.6	53,661	120
Marital status	Married	79.2	273,920	660	41.3	202,215	461
	Widowed	10.9	37,790	144	42.6	208,276	615
	Single/Divorced	9.8	33,975	76	16.1	78,768	186
Ethnicity*	Caucasian	71.0	244,707	630	69.8	341,365	893
	Mulatto/African descent	17.1	59,069	140	15.7	46,567	192
	Other	11.9	41,044	107	14.6	71,327	177
Education	Illiterate. 1–3 years	40.7	140,812	417	48.1	235,534	657
	4–7 years	36.8	127,211	295	36.6	179,189	434
	≥8 years	22.5	77,662	168	15.2	74,536	171
Self-rated health status*	Good	48.5	167,479	645	44.4	216,629	815
	Poor to fair	51.5	178,206	235	55.6	271,149	447
Accidental falls§	Yes	77.7	268,514	645	67.0	327,925	815
	No	22.3	77,171	235	33.0	161,334	447
Number of chronic diseases	None	31.1	103,077	257	26.7	124,555	312
	1	35.5	117,559	285	32.4	150,898	372
	≥2	33.4	110,594	294	40.9	190,924	509
Tobacco*	Nonsmoker	26.2	90,416	237	70.5	344,889	918
	Ex-smoker	51.3	177,276	475	18.4	90,076	221
	Current smoker	22.6	77,993	168	11.1	54,173	122
Alcohol consumption	<1 day/week	65.3	225,640	601	89.8	439,470	1,150
in previous 3 months	1 to 3 days a week	19.6	67,873	156	8.3	40,600	90
	≥4 days/week	15.1	52,172	123	1.9	9,189	22
**TOTAL**		**41**.**4**	**345**,**685**	**880**	**58.6**	**489**,**259**	**1**,**261**

^#^ Weighted proportions in percentage;

^&^ Weighted sample size. Data were weighted to be representative of the elderly population of São Paulo based on the 2000 Census. Brazil.

Sample size; income was categorized by quartiles;

1 to 4 missing responses for these variables;

Accidental falls in previous 12 months.

Table 2 displays the weighted distribution of alcohol intake in the previous three months, according to baseline characteristics and gender. 85.6% of women drank less than once a week, 12.0% drank one to three times a week and 2.4% drank four or more times a week. The moderate intake women were younger and more frequent in higher income and education groups, with good self-rated health status. For men, the alcohol consumption was higher: 19.6% drank one to three times a week and 15.1% drank four or more times a week. In this last group, men were mostly Caucasian, in higher income and education groups, with good self-rated health status and were current smokers.

**Table 2:** Weighted distribution of frequency of alcohol intake in previous three months according to baseline characteristics and gender in adults aged 60 and older: 2000 Saúde. Bem-Estar e Envelhecimento (SABE [Health. Wellbeing and Aging]) Survey.

		MEN				WOMEN			
BASELINECHARACTERISTICS		lowintake&	moderateintake&	highintake&	p-value#	lowintake&	moderateintake&	highintake&	p-value#
		(n = 601)$	(n = 156)$	(n = 123)$		(n = 1,150)$	(n = 90)$	(n = 22)$	
Age	60–64	62.1	19.7	18.2	0.085	85.6	12.0	2.4	0.005
	65–74	64.0	21.7	14.3		90.8	7.6	1.6	
	≥75	74.1	14.3	11.6		93.4	4.9	1.7	
Income	1st quartile	72.2	12.9	14.9		94.3	4.7	1.0	
	2nd quartile	68.5	16.2	15.3	0.008	87.9	12.1	0.0	<0.001
	3rd quartile	67.0	17.9	15.1		85.6	10.9	3.5	
	4th quartile	54.1	33.7	12.2		79.4	15.6	5.1	
Marital status	Married	63.9	21.4	14.6		89.5	8.9	1.6	
	Widowed	68.8	12.3	18.9	0.250	90.4	7.0	2.7	0.354
	Single/Divorced	71.8	13.5	14.7		89.1	10.3	0.7	
Ethnicity*	Caucasian	62.7	22.1	15.3	0.047	89.2	9.0	1.8	0.204
	Mulatto/African descent	77.2	11.7	11.1		94.1	3.4	2.5	
	Other	63.4	16.8	19.8		88.1	10.0	1.8	
Education	Illiterate. 1–3 years	75.6	13.1	11.3		93.4	5.3	1.3	
	4–7 years	60.9	19.7	19.4	<0.001	88.4	10.6	1.0	<0.001
	≥8 years	53.7	31.4	14.9		82.0	12.2	5.8	
Self-rated health status*	Good	56.5	26.5	16.9	<0.001	84.6	12.9	2.5	<0.001
	Poor to fair	73.5	13.1	13.3		93.9	4.7	1.4	
Accidental falls§	Yes	64.2	19.9	15.9	0.528	89.7	8.6	1.8	0.830
	No	69.2	18.6	12.3		90.2	7.8	2.1	
Number of chronicdiseases	None	61.4	18.8	19.8		87.1	10.6	2.3	
	1	61.7	21.2	17.1	0.067	88.2	9.1	2.7	0.083
	≥2	71.8	18.7	9.5		93.2	5.9	0.9	
Tobacco*	Nonsmoker	71.7	19.1	9.3		90.7	7.8	1.5	
	Ex-smoker	65.9	20.9	13.2	0.002	90.1	8.4	1.6	0.075
	Current smoker	56.5	17.3	26.2		83.8	11.3	4.9	
**TOTAL**		**65**.**3**	**19**.**6**	**15**.**1**		**89**.**8**	**8**.**3**	**1**.**9**	

Results presented were weighted to be representative of the elderly population of São Paulo based on the 2000 Census. Brazil.

^&^ Frequency of alcohol intake in previous three months: low intake = < one day a week; moderate intake = one to three days a week; high intake = four or more days a week.

Sample size;

^#^ Rao-Scott chi-square test; income was categorized by quartiles;

1 to 4 missing responses for these variables;

Accidental falls in previous 12 months.

In the multiple polynomial analysis of men, with low intake (less than one day a week) as the reference, moderate intake (1 to 3 days a week) was positively associated with a greater income (OR = 2.53; 95%IC 1.24–5.20) and more years of formal education (OR = 1.73; 95%IC = 1.08–2.76 for those with 4 to 7 years and OR = 2.50; 95%IC = 1.38–4.56 for 8 or more years). High alcohol intake (**≥**4 days a week) was positively associated with a higher level of schooling (OR = 2.12; 95%IC = 1.21–3.70 for 4 to 7 years of education) and current smoking habits (OR = 3.16; 95%IC = 1.42–7.07) and was negatively associated with having two or more chronic diseases (OR = 0.44; 95%IC = 0.23–0.86). In the women group, moderate alcohol consumption was negatively associated with older age (OR = 0.46; 95%IC = 0.26–0.79 for 65 to 74 years and OR = 0.43; 95%IC = 0.21–0.87 for 75 years of age or older) and high alcohol intake with poor to fair self-rated health status (OR = 0.17–95%IC = 0.04–0.68).

Table 3 and Table 4 display the alcohol use in 2006 in relation to the reported use in 2000 according to baseline characteristics and gender. 17.0% of men and 10.2% of women increased the alcohol consumption between years. Both men and women who increased their use with time were more frequently in higher education groups and in the group of those who considered themselves in good health status. Women were also more frequently older and had a history of fall in the last 12 months. The associations of increase in alcohol consumption and baseline characteristics in the final model of multiple polynomial analyses are shown in Table 5. For men, increased consumptions were negatively associated with poor to fair self-rated status and for women, were positively associated with a higher level of schooling and was negatively associated with former smoker habits. Stable consumption was positively associated with income and higher education for both men and women and with age for men.

**Table 3:** Weighted distribution of frequency of alcohol intake of men in previous three months in 2006 in relation to use reported at baseline (2000) according to baseline characteristics: 2000/2006 *Saúde, Bem-Estar e Envelhecimento* (SABE [Health, Wellbeing and Aging]) Survey.

		MEN (n = 398)$				
BASELINE CHARACTERISTICS		Stable lowdrinkers&	Decreasingdrinkers*	Increasingdrinkers*	Stabledrinkers¥	p-value#
		(n = 225)	(n = 70)	(n = 55)	(n = 48)	
Age	60–64	44.7	16.2	22.6	16.5	0.101
	65–74	55.8	16.4	13.8	14.0	
	≥75	63.6	20.0	8.6	7.8	
Income	1st quartile	56.6	19.3	20.0	4.1	0.081
	2nd quartile	61.0	14.5	9.8	14.7	
	3rd quartile	55.0	13.0	18.5	13.5	
	4th quartile	38.5	19.5	17.3	24.7	
Marital status	Married	50.2	17.2	17.6	15.0	0.592
	Widowed	56.0	23.4	13.3	7.3	
	Single/Divorced	64.4	6.0	15.2	14.6	
Ethnicity	Caucasian	50.4	17.4	16.1	16.1	0.461
	Mulatto/African descent	63.8	10.3	17.9	8.0	
	Other	45.6	21.0	20.2	13.2	
Education	Illiterate, 1–3 years	62.4	14.1	16.4	7.1	0.003
	4–7 years	49.7	20.8	15.8	13.7	
	≥8 years	37.4	14.4	19.9	28.6	
Self-rated health status	Good	42.1	18.0	21.0	18.9	0.007
	Poor to fair	62.4	15.3	12.7	9.5	
Accidental falls§	Yes	51.9	16.4	17.8	13.8	0.820
	No	52.3	18.1	13.2	16.4	
Number of chronic diseases	None	48.4	20.7	17.8	13.1	0.351
	1	46.4	15.6	18.6	19.4	
	≥2	61.5	12.9	15.6	10.0	
Tobacco	Nonsmoker	58.0	13.1	15.7	13.3	0.265
	Ex-smoker	51.4	14.4	18.2	16.0	
	Current smoker	44.0	29.0	15.7	11.3	
**TOTAL**		**51**.**0**	**16**.**7**	**17**.**0**	**14**.**3**	

Results presented were weighted to be representative of the elderly population of São Paulo based on the 2000 Census. Brazil;

Sample size;

^&^ less than 1 day per week in 2000 and in 2006;

in 2006 in relation to intake in 2000;

once a week or more in 2000 and in 2006;

^#^ Rao-Scott chi-square test; income was categorized by quartiles;

Accidental falls in previous 12 months.

**Table 4:** Weighted distribution of frequency of alcohol intake of women in previous three months in 2006 in relation to use reported at baseline (2000) according to baseline characteristics: 2000/2006 *Saúde, Bem-Estar e Envelhecimento* (SABE [Health, Wellbeing and Aging]) Survey.

		WOMEN (n = 696)$				
BASELINE CHARACTERISTICS		Stable lowdrinkers&	Decreasingdrinkers*	Increasingdrinkers*	Stabledrinkers¥	p-value#
		(n = 563)	(n = 31)	(n = 72)	(n = 30)	
Age	60–64	73.9	6.4	11.2	8.5	0.022
	65–74	83.3	3.3	10.3	3.2	
	≥75	83.6	5.6	8.0	2.8	
Income	1^st^ quartile	85.0	4.6	8.6	1.9	0.091
	2^nd^ quartile	77.1	5.2	12.8	4.9	
	3^rd^ quartile	74.1	7.6	12.5	5.9	
	4th quartile	69.3	6.8	12.2	11.7	
Marital status	Married	78.1	4.5	11.1	6.3	0.669
	Widowed	81.3	5.7	9.6	3.4	
	Single/Divorced	82.1	3.2	9.0	5.7	
Ethnicity	Caucasian	79.4	5.0	10.1	5.5	0.722
	Mulatto/African descent	85.1	2.0	9.9	3.0	
	Other	76.6	7.1	11.2	5.1	
Education	Illiterate, 1–3 years	88.6	4.3	4.6	2.5	<0.001
	4–7 years	76.5	4.0	12.3	7.2	
	≥8 years	59.7	8.7	23.8	7.8	
Self-rated health status	Good	73.1	8.2	11.1	7.6	0.002
	Poor to fair	85.3	2.1	9.6	3.0	
Accidental falls§	Yes	81.0	3.2	10.5	5.3	0.028
	No	77.4	8.6	9.6	4.5	
Number of chronic diseases	None	74.3	6.9	10.9	7.9	0.186
	1	81.5	3.8	9.2	5.6	
	≥2	83.8	3.2	10.5	2.5	
Tobacco**	Nonsmoker	79.1	4.1	11.9	4.9	0.061
	Ex-smoker	87.1	4.1	2.3	6.5	
	Current smoker	74.7	10.3	10.8	4.2	
**TOTAL**		**79**.**9**	**4**.**8**	**10**.**2**	**5**.**1**	

Results presented were weighted to be representative of the elderly population of São Paulo based on the 2000 Census. Brazil;

Sample size;

^&^ less than 1 day per week in 2000 and in 2006;

in 2006 in relation to intake in 2000;

once a week or more in 2000 and in 2006;

^#^ Rao-Scott chi-square test; income was categorized by quartiles;

Accidental falls in previous 12 months;

10 missing responses for these variables.

**Table 5:** Association between alcohol intake of participants evaluated in 2006 in relation to use reported in baseline study (2000) according to baseline characteristics: 2000/2006 Saúde, Bem-Estar e Envelhecimento (SABE [Health, Wellbeing and Aging]) Survey.

		MEN		WOMEN	
BASELINE CHARACTERISTICS		Increasing	Stable	Increasing	Stable
		Drinkers&	drinkers¥	drinkers&	drinkers¥
Age	60–64	1	1	1	1
	65–74	0.83(0.40–1.75)	1.60(0.61–4.20)	1.31(0.55–3.14)	0.40(0.12–1.33)
	≥75	1.13(0.39–3.33)	3.71(1.20–11.9)*	1.07(0.39–2.90)	0.55(016–1.86)
Income	1st quartile	1	1	1	1
	2nd quartile	0.40(0.10–1.61)	2.91(0.78–10.9)	1.31(0.47–3.63)	2.36(0.44–12.52)
	3rd quartile	0.86(0.30–2.50)	2.78(0.59–12.90)	1.25(0.48–3.27)	3.20(0.68–14.99)
	4th quartile	0.90(0.26–3.11)	4.40(1.10–16.70)*	1.19(0.44–3.24)	4.85(1.08–21.83)*
Education	Illiterate, 1–3 years	1	1	1	1
	4–7 years	0.84(0.42–1.75)	1.60(0.61–4.20)	3.26(1.83–5.81)*	4.11(1.03–16.27)*
	≥8 years	1.14(0.39–3.32)	3.71(1.16–11.9)*	6.52(2.58–16.15)*	3.11(0.57–16.99)
Self-rated health status	Good	1	1		
	Poor to fair	0.40(0.17–0.89)*	0.52(0.21–1.34)		
Tobacco	Nonsmoker			1	1
	Ex-smoker			0.25(0.06–0.99)*	1.65(0.48–5.67)
	Current smoker			1.41(0.56–3.50)	2.04(0.32–13.05)

Data weighted to be representative of the elderly population of São Paulo based on the 2000 Census, Brazil. The frequency of alcohol intake in 2006 was compared with the one referred in 2000, with alcohol intake stable low drinkers (less than 1 day per week in both periods) as reference.

^&^ higher intake in 2006 in relation to the referred in 2000;

once a week or more in 2000 and in 2006;

variable statistically significant in the final model of multiple polynomial analysis; Income was categorized by quartiles.

For the evaluation of alcohol use by age in the two different cohorts with a six-year difference in birth dates, the frequency of alcohol intake among individuals in the same age groups in 2000 and 2006 was compared. The predominant tendency was toward an increase in the frequency of moderate intake (1 to 3 days a week), which occurred in all age groups analyzed (cohort effect) for both sex. High alcohol intake (4 or more days a week) was lowest among individuals aged 75 years or older (Table 6).

**Table 6:** Weighted distribution of frequencies of alcohol intake in previous three months according to age group in 2000 and 2006 (cohort effect) by gender: 2000/2006 *Saúde, Bem-Estar e Envelhecimento* (SABE [Health, Wellbeing and Aging]) Survey.

			2000*			2006$		
SEX			low intake&	moderate intake&	high intake&	low intake&	moderate intake&	high intake&
			%(95% CI)#	%(95% CI)	%(95% CI)	%(95% CI)	%(95% CI)	%(95% CI)
Men	Age	60–64	62.1(54.9–68.8)	19.7(13.9–27.2)	18.2(13.4–24.2)	–	–	–
		65–74	64.0(57.9–69.7)	21.7(16.9–27.5)	14.3(10.7–18.7)	56.3(45.0–66.9)	31.7(22.8–42.2)	12.0(6.1–22.3)
		>75	74.1(68.7–78.9)	14.3(10.9–18.4)	11.6(9.1–14.7)	69.1(60.1–76.8)	19.7(13.2–28.4)	11.2(6.9–17.8)
Women	Age	60–64	85.6(80.9–89.3)	12.0(8.4–16.7)	2.4(1.1–4.9)	–	–	–
		65–74	90.8(87.3–93.4)	7.6(5.5–10.5)	1.6(0.8–3.2)	87.5(82.3–91.3)	11.7(7.9–16.9)	0.8(0.2–2.7)
		>75	93.4(90.4–95.5)	4.9(3.0–7.9)	1.7(0.9–3.4)	92.2(87.8–95.1)	6.4(3.8–10.6)	1.4(0.5–3.8)

Weighted by weights from year 2000;

Weighted by weights from year 2006.

^&^ Frequency of alcohol intake in previous three months: low intake = <one day a week; moderate intake = one to three days a week; high intake = four or more days a week;

^#^ CI = confidence interval.

To explore the pattern of attrition, individuals lost to follow up due to refusal, being lost or to death were compared to those included in the 2006 evaluation. The former group was older (27.7% aged 75 years or older vs. 17.4% those included in the second wave, p<0.001), had a greater frequency of men (44.2% vs. 39.2%, p = 0.014) and the individuals in this group were more likely to have two or more chronic diseases (42.2% vs. 34.4%, p = 0.007). Moderate alcohol intake (1 to 3 times a week) was 11.2% vs. 14.4% and high intake (≥4 times a week) was 8.9% vs. 6.1%, respectively, in the two groups (p<0.001). No differences were found regarding the other variables evaluated.

## Discussion

The frequency of alcohol intake among the older adults of São Paulo was low, which is in agreement with findings reported in studies involving other populations of older adults in the world [6]–[8]. As reported in the literature, moderate and high alcohol intake was strongly associated with the male gender and a higher income [6], [8], [10]. Moderate intake was greater among married individuals and those with a higher level of schooling and smoking was associated with greater alcohol intake [6], [10]. These socio-demographic characteristics are similar to those described for other populations, such as those of the United States of America and Europe [8], [10], [11], [23], [24].

Recently, Kirchner et al. (2007) found that individuals aged 65 to 74 years are more prone to alcohol use than those aged 75 years or older [9]. The same finding is reported in the present study, which showed a reduction in alcohol use with the increase in age in both the baseline and follow-up periods. The longitudinal evaluation of alcohol intake revealed that both individuals who reported higher consumption or stable more than once a week consumption were mainly between 60 and 64 years of age, with a significant difference in relation to the frequencies found in individuals aged 75 years or older [6], [8]. These findings are similar to those described in other studies on alcohol use in the city of São Paulo, which report a decline in the occurrence of problems related to substance use in older cohorts [16], [17], [25], [26]. Moore et al. (2005) reports a similar observation with regard to older Americans [8].

According to Newsom et al. (2012), older adults tend to change their habits after receiving information on chronic health conditions [27]. The present results seem to corroborate this statement, as moderate alcohol use occurred mainly among those who considered themselves to be in good health (data not shown). Moreover, a lower frequency of high alcohol intake (four or more times a week) was found among individuals with two or more chronic diseases. Self-rated health has been used as a tool for the understanding of older adults with regard to their clinical conditions and those with a greater risk of illness and death. In the general population in Sweden, risk factors (excessive drinking and smoking) and uncontrolled health conditions were found to make major contributions to self-rated poor health [11].

Interestingly, an increase in the frequency of moderate alcohol intake (1 to 3 days a week) was found in the follow-up cohort, as well as in the younger cohort of birth date. In recent years, moderate alcohol use has been related to a set of benefits, especially for the cardiovascular system. Thus, the increase in moderate alcohol use among the individuals in comparison to the same age groups at baseline may be related to information regarding such health benefits. This hypothesis should be explored further, as it suggests that older adults in the city of São Paulo may have undergone a change of behavior over time regarding alcohol use, which differs from reports involving other populations.

The present findings allow determining the current behavior of alcohol intake in this representative sample of the population of older adults in the city of São Paulo. The analysis was restricted to the frequency of alcohol intake in the previous three months, which could decrease the memory bias. However, the fact that there was no measurement of the amount of alcohol consumed on each occasion constitutes a limitation of the present study. The period of the year of data collection in the follow-up was not the same as in baseline, and this could introduce a bias in the analysis of change of alcohol intake, since in some periods of the year, such as festivities, the alcohol intake is more likely to be higher. Moreover, there was no information on lifetime alcohol use, which impedes the determination of problems related to chronic alcohol use. The individuals identified in the study as non-drinkers may actually be a combination of those who never drank and those who stopped drinking. It is also possible that alcohol intake had been underestimated, as the data were obtained from self-reports and some individuals may consider drinking to be shameful behavior.

The longitudinal evaluation was carried out with 52% of the individuals analyzed in 2000. Among the total sample, 30% died in the follow-up period. The attrition group was older and more likely to have more chronic diseases. This group was also more likely to use alcohol four or more times a week, however, the differences in intake frequency in comparison to the group included in the follow-up analysis do not explain the findings of the study. A selection bias in the follow up information may be present, as data were collected in a selected sample of those who survived in this period.

In conclusion, the older adults in the present population-based sample from the city of São Paulo exhibited a low frequency of alcohol intake, which is similar to findings reported for other samples of older adults surveyed throughout the world. Men presented higher alcohol consumption than women. Individuals who consumed alcohol generally had good self-perceived health, higher income and a higher level of schooling, as reported in studies carried out in other countries [6], [7], [8], [11]. In follow up, men with good self-rated health status and women with higher education were associated with increase in intake of alcohol with time. For both men and women, higher income and education were positively associated with stable drinking with time. The comparison among different cohorts of birth date showed a tendency to increase in moderate alcohol consumption in recent years. Despite the low frequency of alcohol intake observed, about one fourth of the men and 15% of women were stable drinkers or referred an increase in intake in the follow up.The assessment of baseline characteristics associated with drinking patterns and changes in alcohol intake with ageing and time provides assistance for the formulation of appropriate public policies for this age group of the population. The results suggest that such policies should be directed to the ageing population, as it may be difficult to identified those who are likely to change their alcohol intake.
